# Fractal properties of shoreline changes on a storm-exposed island

**DOI:** 10.1038/s41598-017-08924-9

**Published:** 2017-08-15

**Authors:** Xiaojing Zhong, Peng Yu, Shenliang Chen

**Affiliations:** State Key Laboratory of Estuarine and Coastal Research, East China Normal University, 3663 North Zhongshan Road, Shanghai, 200062 China

## Abstract

Extreme storm events and their consequent shoreline changes are of great importance for understanding coastal evolution and assessing storm hazards. This work investigates the fractal properties of the spatial distributions of shoreline changes caused by storms. Wavelet analysis and upper-truncated power law (UTPL) fitting are used to study the power spectra of shoreline changes and to evaluate the upper limits of the cross-shore erosion and accretion. During a period affected by storms, the alongshore shoreline change patterns are strong on the 15 km scale but are weak with lower spectral power on the 20 km scale. The areas adjacent to the eroded shoreline are usually accrete, and the cross-shore extent of erosion is larger than that of accretion when the coast is affected by storms. The fractal properties of shoreline changes due to storms are found to be temporally continuous: the effects of later storms build on the preceding shoreline conditions, including both the effects of previous storms and the subsequent shoreline recoveries. This work provides a new perspective on the various scales of the spatial variations of the morphodynamics of storm-affected shorelines.

## Introduction

Fractals are defined as irregular and fragmentary forms, usually exhibiting self-similar patterns^1, 2^. Examples of fractals are common in nature and include coastlines^3, 4^, river networks^5^ and earthquakes^6, 7^. Fractals exist not only in spatial patterns but also in the time series of physical processes^8–11^. How fractals are generated and interact remains a topic of interest in the study of natural phenomena. However, the fractal properties of different phenomena can reveal their spatial and temporal scaling characteristics and may help identify the internal mechanisms and possible trends of these phenomena^12–16^.

Although there is currently no strict definition of a “fractal”, a general set of features is used to characterize fractals^1, 2, 8, 17^, the most notable of which is the “fractal dimension”. The fractal dimension measures the ratio between the complexity of a fractal pattern and its corresponding scale^1, 17^. For multifractal patterns, the multifractal dimension is a continuous spectrum of exponents that represents the variation of the scaling behaviours in different parts of the fractal pattern^18^. The mathematical basis of a fractal dimension (*D*) is the power-law relationship between the number of segments (*N*) and the measurement scale (*r*)^1^:

1 $$N\propto {r}^{-D},$$

or, for times series, between the power-spectral density (*S*) and frequency (*f*)^19^:

2 $$S\propto {f}^{-\beta }.$$

However, the power-law relationship is not strictly obeyed by natural phenomena^20–23^; for instance, patterns may be pronounced at some scales^12, 24, 25^, and there is usually an upper/lower limit to the scaling of the pattern^26, 27^. In the first case, spectral analysis can detect the intensities of patterns at different scales and reveal the dominant pattern and its scale, when it exists. For the latter case, an upper-truncated power law (UTPL) introduced by Burroughs & Tebbens^28^ can be used to estimate the fractal dimension (*D*) and upper limitation of object size (*r*_*T*_):

3 $$N(r)=C({r}^{-D}-{r}_{T}^{-D}),$$

where *N*(*r*) is the cumulative number of objects with characteristic sizes greater than or equal to *r* and *C* is a constant. Data sampling limitations and changes in the studied physical processes are considered potential causes of this upper truncation^28^, which reduces the cumulative number for each object size^29^.

In the decades following the seminal work by Mandelbrot^3^ that connected fractal properties to coastline variations, cross-disciplinary research on the topic, including work in fractal geometry, geophysics and geomorphology, has flourished^30–33^. A growing body of evidence has been presented in support of the view that shoreline movement is an irregular fractal with statistically self-similar spatial and temporal characteristics^29, 34^. Sandy coasts exhibit large variations in relatively short periods and are sensitive to dynamic conditions, such as wind and waves, making them an ideal subject for studying fractal geometry. Knowing the fractal properties of sandy coasts could further our understanding of shoreline morphodynamics.

Based on the extensive bathymetric dataset at Duck Site, North Carolina, Southgate & Moller^35^ divided the retrieved cross-shore profiles according to their Hurst exponents. Fractal morphodynamics were found at time intervals with weak to moderate wave conditions. Instead of beach profile changes, Tebbens *et al*.^29^ focused on the horizontal, shore-perpendicular change in the shoreline position measured in tens of kilometres along the northern Outer Banks of North Carolina, United States. Based on wavelet analysis of the shoreline change, they were able to demonstrate that the shoreline change is self-affine with a scaling exponent that varies from 1.2 to 2.1, indicating that the shoreline change is non-stationary with a long-range persistence. By applying UTPL to the data for one section of coast, they found that the upper limits of the maximum shore-perpendicular erosion and accretion were 25 m and 11 m, respectively, during the study period, and the upper limit of the maximum continuous alongshore erosion or accretion was 7 km. Further studies by Lazarus *et al*.^34, 36^ extended the fractal scale up to 10 km, depending on the considered data site and period. These findings are important as they imply that the cumulative shoreline variations over a period of a year or a few months can have spatial scales that appear to be unrelated to the scales of the external forcings.

Previous studies have typically focused on the general fractal properties of coastal morphology over multiyear time periods with relatively low temporal resolutions and have paid little attention to these properties in extreme conditions, such as storms. To what extent such an event-scale process affects general coastal evolution remains an open question. More studies involving different coastal settings and larger spatial scales are clearly required.

In this work, we analyse shoreline change data collected from 7 beach surveys conducted over a 3-year period along the shoreline around Hainan Island, China. The main aim of this paper is to quantify the spatial characteristics of shoreline response to storms using wavelet and spectral analysis methods and the UTPL curve fitting. Spectral analysis is used to study the self-affine behaviour of shoreline change in the alongshore direction, and UTPL is applied to identify the cross-shore characteristics of shoreline movement.

### Study area and field surveys

**Figure 1:**
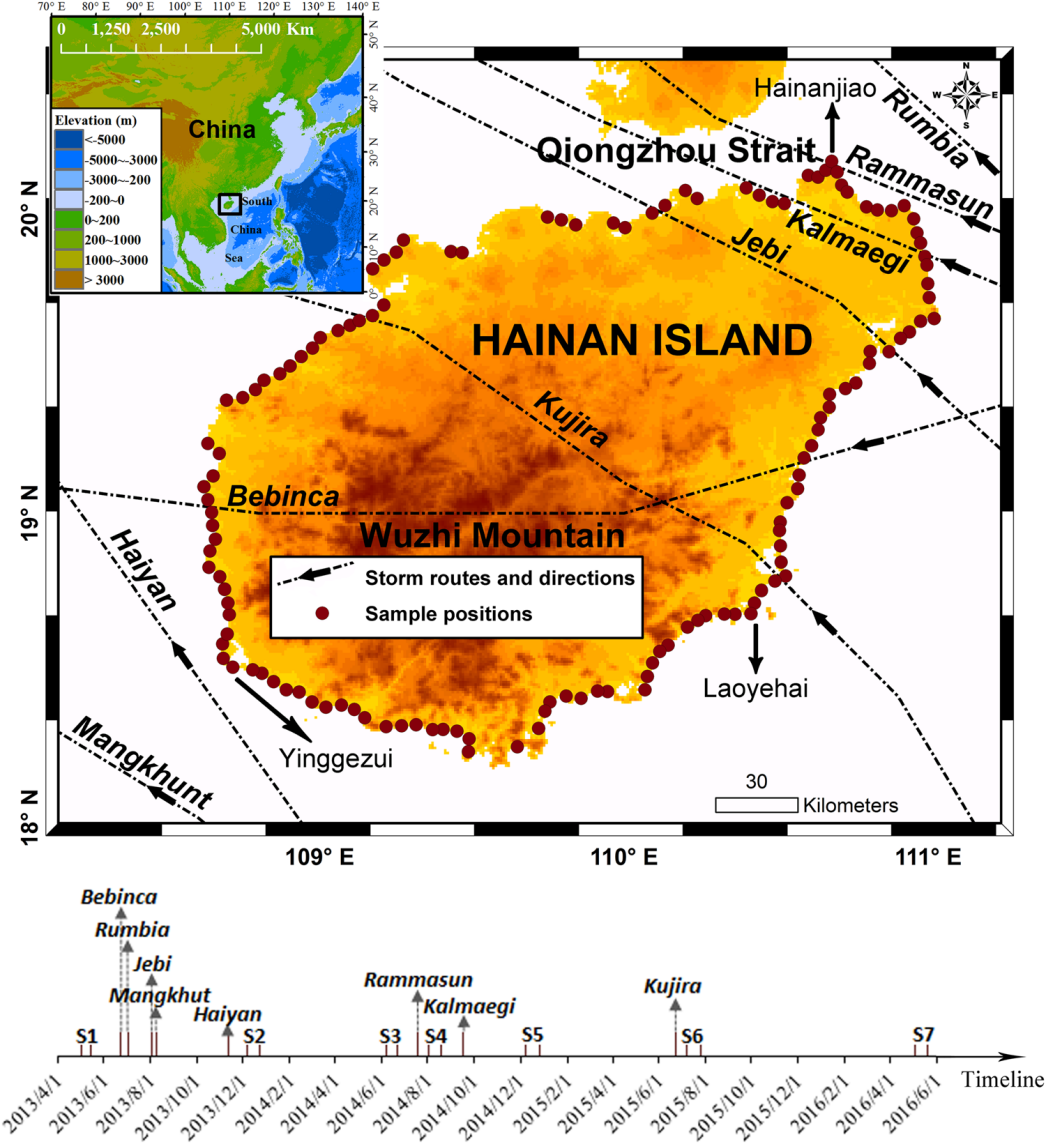
Map of Hainan Island, sample positions, storms during the surveys and their tracks. Topographic and bathymetric data are from GEBCO (http://www.gebco.net/), and the storm routes are the best tracks from CMA^47^ (http://tcdata.typhoon.gov.cn/). The maps are generated using ArcMap [10.1] (http://www.esri.com/). In the timeline, S1 to S7 refer to the seven field surveys.

Hainan Island is located in the South China Sea (Fig. 1) and has an area of approximately 33,907 km^2^ and a shoreline length of over 1,400 km^37^. The highest mountain on the island, Wuzhi Mountain, from which most of the rivers on Hainan Island originate and flow to the coastal zone, is in the central area of the island. Hainan Island was formed with the opening of Qiongzhou Strait at 8.5 ka B.P.^38, 39^. Until 2.0 ka B.P., many sedimentary systems formed in the coastal area, including sandy beaches, lagoons, mangrove wetlands and coral reefs^40^. Since then, the sediment movements around Hainan Island have been dominated by waves and typhoon-induced storms, and the sediments involved in coastal evolution now originate from the resuspension of deposited sediment and backshore erosion^41^. Sandy coasts are the main shoreline types, while headlands at Hainanjiao (HNJ), Laoyehai (LYH) and Yinggezui (YGZ) protrude from the shoreline and divide the coast into three sections. The east coast, with an eastward-facing shoreline, is from HNJ to LYH and is mainly formed of lagoons, tidal channels and sand spits. The south coast, with a primarily southward-facing shoreline, is from LYH down to YGZ and has more offshore islands and beach rocks than that of the east coast. The tidal range on the west and north coasts exceeds 1.2 m, while on the east and south coasts, it is less than 1 m^41^. The climate of the Hainan Island and the South China Sea is dominated by the East Asian monsoon, with northwest winds in winter and south and southeast winds in summer^41, 42^. Seasonal changes in winds influence the surface current in the South China Sea^43^. The mean flow direction around Hainan Island from October to March is southwestward, and it shifts to northeastward between April and September^44, 45^. The direction and energy of surface waves around the island are closely correlated with the seasonal wind direction and forcing strength^41^. Hainan’s coast is often affected by large storms that are caused by tropical cyclones, which occur with an average frequency of approximately 2.5 times per year^46^.

To monitor shoreline change around Hainan Island, 132 sampling profiles encompassing all of the beaches around the island were measured at spatial intervals of approximately 5 km (Fig. 1). Beach-level measurements were collected seven times with the Trimble Real Time Kinematic-Global Positioning System (RTK-GPS) (Fig. 1 and Table 1); based on the seven surveys, we obtained six sets of shoreline change data, which are consistent with the shoreline changes at the sampling: S1-S2, S2-S3, S3-S4, S4-S5, S5-S6 and S6-S7. The last storm that passed by Hainan Island before our surveys was Son-tinh^47^, which occurred in late October 2012. Between our first survey S1 (in May 2013) and last survey S7 (in May 2016), there were 8 storms that affected Hainan, and their meteorological data are listed in Table 1. The east and south coasts, from HNJ clockwise to YGZ, face the storms directly (Fig. 1). During the 6 time periods between the surveys, S2-S3 and S6-S7 were not affected by storms, while S1-S2 was exposed to 5 storms; for the other three periods, the shoreline changes were affected by a single storm.

**Table 1:** Dates of the seven surveys and the meteorological data of the storms that affected Hainan Island during the surveys.

Survey/*Storm*	Start date	End date	International number ID	Minimum central pressure (hPa)	Maximum sustained wind speed (kt)	Average radius of 30 kt winds or greater (nm)	Grade
**S1**	**2013/4/30**	**2013/5/14**					
***Bebinca***	2013/6/19	2013/6/24	1305	990	40	135	3
***Rumbia***	2013/6/27	2013/7/2	1306	985	50	165	4
***Jebi***	2013/7/28	2013/8/3	1309	985	50	200	4
***Mangkhut***	2013/8/5	2013/8/8	1310	992	40	120	3
***Haiyan***	2013/11/3	2013/11/11	1330	895	125	225	5
**S2**	**2013/12/6**	**2013/12/21**					
**S3**	**2014/6/6**	**2014/6/20**					
***Rammasun***	2014/7/9	2014/7/20	1409	935	90	160	5
**S4**	**2014/8/1**	**2014/8/17**					
***Kalmaegi***	2014/9/11	2014/9/17	1415	960	75	305	5
**S5**	**2014/12/6**	**2014/12/24**					
***Kujira***	2015/6/19	2015/6/25	1508	985	45	110	3
**S6**	**2015/7/7**	**2015/7/24**					
**S7**	**2016/5/2**	**2016/5/19**					

The meteorological data were acquired from the Regional Specialized Meteorological Center (RSMC) Tokyo-Typhoon Center (http://www.jma.go.jp/jma/indexe.html). The categories of tropical cyclone intensities are designated by the Japan Meteorological Agency: Grade 3 is a tropical storm, Grade 4 is a severe tropical storm, and Grade 5 is a typhoon.

## Results

**Figure 2:**
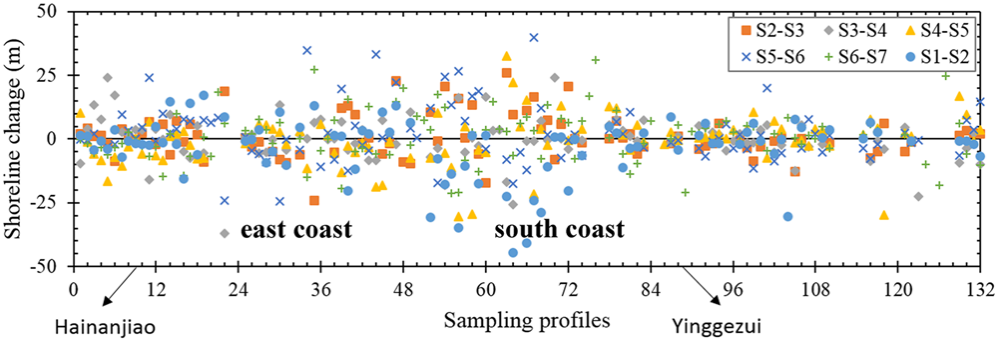
Horizontal changes in the shoreline positions for the shore-perpendicular profiles shown in Fig. 1 during the 6 survey periods. Positive values represent accretion, negative values represent erosion.

Shoreline change is measured by determining the horizontal change in the position of the 0-m contour sampled from the shore-perpendicular profiles; the measured shoreline change data are plotted in Fig. 2. For the six survey periods, the shoreline movements always consisted of both accretion and erosion. Thus, whether a storm occurs or not, erosion occurs somewhere along the shoreline. In addition, the shoreline could locally move seaward even when influenced by a storm. The shoreline changes of the profiles along the east and south coasts are conspicuously more dispersive than those of the other coasts, indicating that shoreline movements between HNJ and YGZ are more active because this area directly faces the storms. Therefore, shoreline movements on the profiles from HNJ clockwise to YGZ are used to analyse the fractal properties of shoreline changes under the influence of storms.

### Alongshore fractal patterns of shoreline change

**Figure 3:**
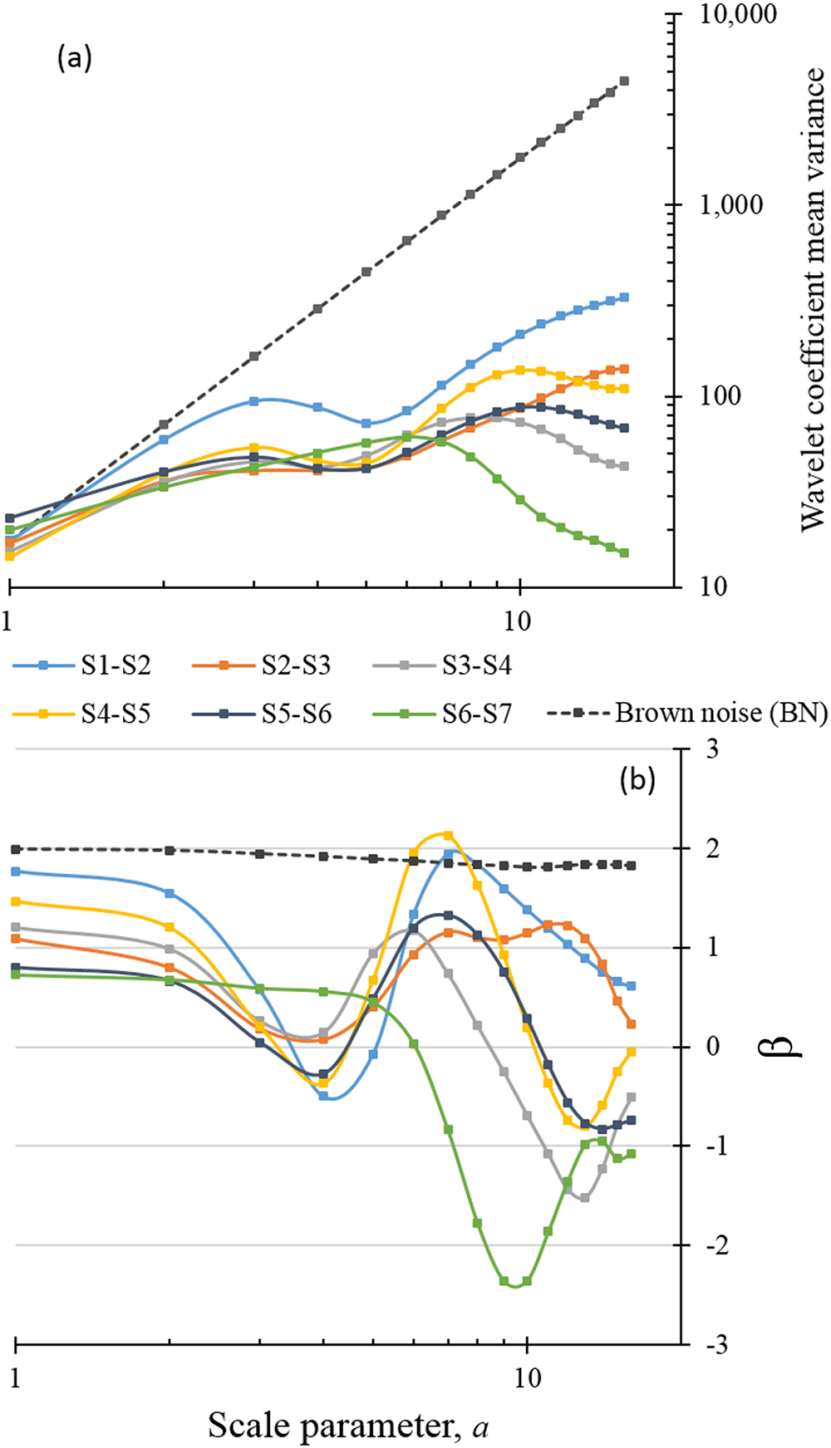
Wavelet transform results of the brown noise and shoreline changes on the eastern and southern coasts, from HNJ to YGZ. (**a**) Log-log plots of the power-spectral density and scale (~1/frequency). (**b**) Power-law relationships between the power-spectral density and scale; β is calculated from the data points in (**a**).

By applying a wavelet transform to the alongshore shoreline changes, both the dominant patterns of variability and how these patterns vary in space can be determined. Figure 3 presents the relationships between the power-spectral densities and the alongshore scales of shoreline changes, in which the wavelet coefficient mean variance (WCMV) is the power spectrum of the shoreline change, *a* is the scaling parameter of the wavelet transform, which varies from 1 to 16, and *β* is the power spectral exponent.

In general, the WCMV of the shoreline changes increases along with increased scale, but the power-law relationship with scaling is not strictly obeyed; the increase of the WCMV is non-monotone and breaks off in the scale range of $3\le a\le 5$ (Fig. 3(a)). As expected, the power spectra of S6-S7 are significantly different from those of the other shoreline changes; the power-law relationship with scale is closely followed by WCMV at $1\le a\le 6$, which implies that the shoreline change can be fractal for spatial scales up to 30 km (*a* × 5 km = 30 km (*a* = 6), and 5 km is the distance between the adjacent sampling locations). For larger scales, the trends and magnitudes of the WCMV vary between sampling periods, and there is no clear demarcation of their spatial trends. The relationship between power-spectral densities and the alongshore scales of the shoreline changes is inconclusive for scales larger than 30 km based on the available database. Compared with brown noise (BN), the power-spectral densities of the shoreline changes are lower for larger scales, which demonstrates that the variations in the large-scale shoreline changes are less significant than the small-scale changes. There are two potential reasons for this difference: limitations of the database, e.g., the temporal and spatial scales of the surveys, and large-scale controlling mechanisms that differ from their small-scale equivalents.

The value of *β* measures the persistence of alongshore shoreline changes: on scales of $\beta  > 1$, the correlations of the shoreline changes for the different sampled profiles are considered strong, meaning that shoreline changes can be affected by shoreline changes both nearby and at a distance; for $0 < \beta  < 1$, the correlations of the shoreline change among the sampled positions at distances on a corresponding scale are weak; on scales with $\beta  < 0$, the shoreline change is defined as antipersistent^48^. Based on these results and the fact that shoreline changes are fractal, it can be deduced that the effects of storms on shorelines occur primarily on the scales of 15 km to 25 km (($3\le a\le 5$) × 5 km). More specifically, for a time period affected by a storm, the alongshore pattern of shoreline change is distinctly robust when the scale parameter $a=3$ for the WCMV reaches a peak at this scale; however, patterns with a scale of $4\le a\le 5$ are much less likely to show the expected power-law relationship, followed by fractal shoreline changes. For instance, the shoreline change in S1-S2 is affected by 5 storms within 6 months, which is the highest number among the six survey periods, and thus exhibits the highest WCMV (i.e., *a* = 3). In contrast, the shoreline changes over S6-S7, which spans approximately 10 months without a storm, show no local extreme WCMV in the scale range 3 ≤ *a* ≤ 5. Hence, the series of wavelet coefficients for the scale parameter *a* = 3 should reveal the alongshore patterns of shoreline changes enhanced by storms, while the coefficients for *a* = 5 are relate to patterns damped by storms.

**Figure 4:**
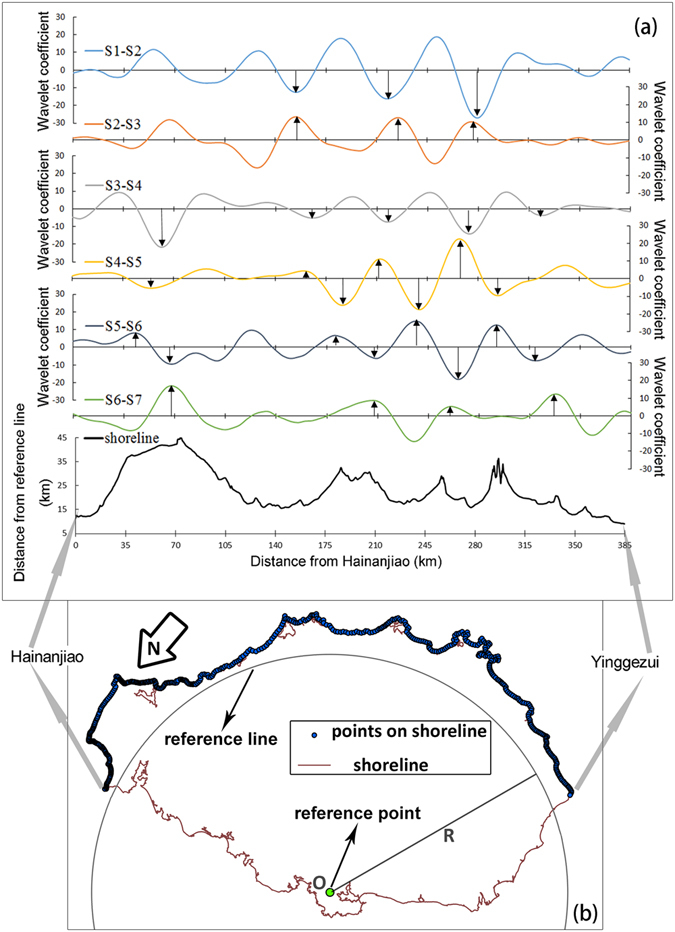
Wavelet coefficients of shoreline change series for the scale parameter a = 3 and a sketch of the coast. The bottom curve in (**a**) is based on (**b**), where the distance from the reference line equals the distance between O and points on the coast line minus the radius of the reference line, R. The position of the reference point, O, is 109.6964°E, 19.3257°N. The adjacent wavelet coefficient series usually present as mirror image of each other, and large amplitudes of these coefficients appear in relatively fixed areas. Arrows within the coefficient curves show the seriously eroded coastal areas after strong storms, which are efficiently recovered afterwards. The map in (**b**) is generated using ArcMap [10.1] (http://www.esri.com/) and the shoreline is based on the topographic and bathymetric data from GEBCO (http://www.gebco.net/).

Figure 4 presents the alongshore fluctuations of wavelet coefficients for scale of *a* = 3, where negative values indicate erosion of the coast and positive values indicate accretion at the coasts, based on the transformed shoreline changes. The wavelet coefficients show considerable variations, and distinct valleys usually appear at the coast closest to the landing position of a storm, e.g., the largest erosion during S3-S4 is located on the northeast coast (approximately 40~80 km from HNJ), where the brunt of storm Rammasun struck land, manifesting onshore storm impact of the aforementioned scale. Due to the strongest patterns affected by the storm, wavelet coefficients fluctuate as distinct units, with alongshore scales of approximately 30 km. The relationship between the scale parameter *a* and the fluctuation units of the shoreline is established through an alongshore scale of *a* × 4 km (sampling interval), which indicates the distance from the coast with a wavelet coefficient of zero to the nearest position with a local extreme wavelet coefficient. Valleys (erosion) of wavelet coefficients are always accompanied by crests (accretion) on either side, indicating the synergistic effect of shoreline changes in the alongshore direction at spatial scales over 30 km. Furthermore, there exist distinct contrasts between the wavelet coefficient series of the adjacent survey periods: for units with strong erosion during storm-affected periods, a certain amount of accretion often followed the storm, and vice versa. This reveals several properties of storm-exposed shoreline movements: the time and place of the erosion and accretion events must be both random and known; for the eastern and southern coasts of Hainan Island, storm-induced erosion and accretion did not continuously occur at a specific location over the entire survey period. Thus, although the shoreline fluctuates dramatically when forced by a storm, the shoreline of Hainan Island as a whole is still at dynamic equilibrium.

### Upper-truncation of shoreline changes

**Figure 5:**
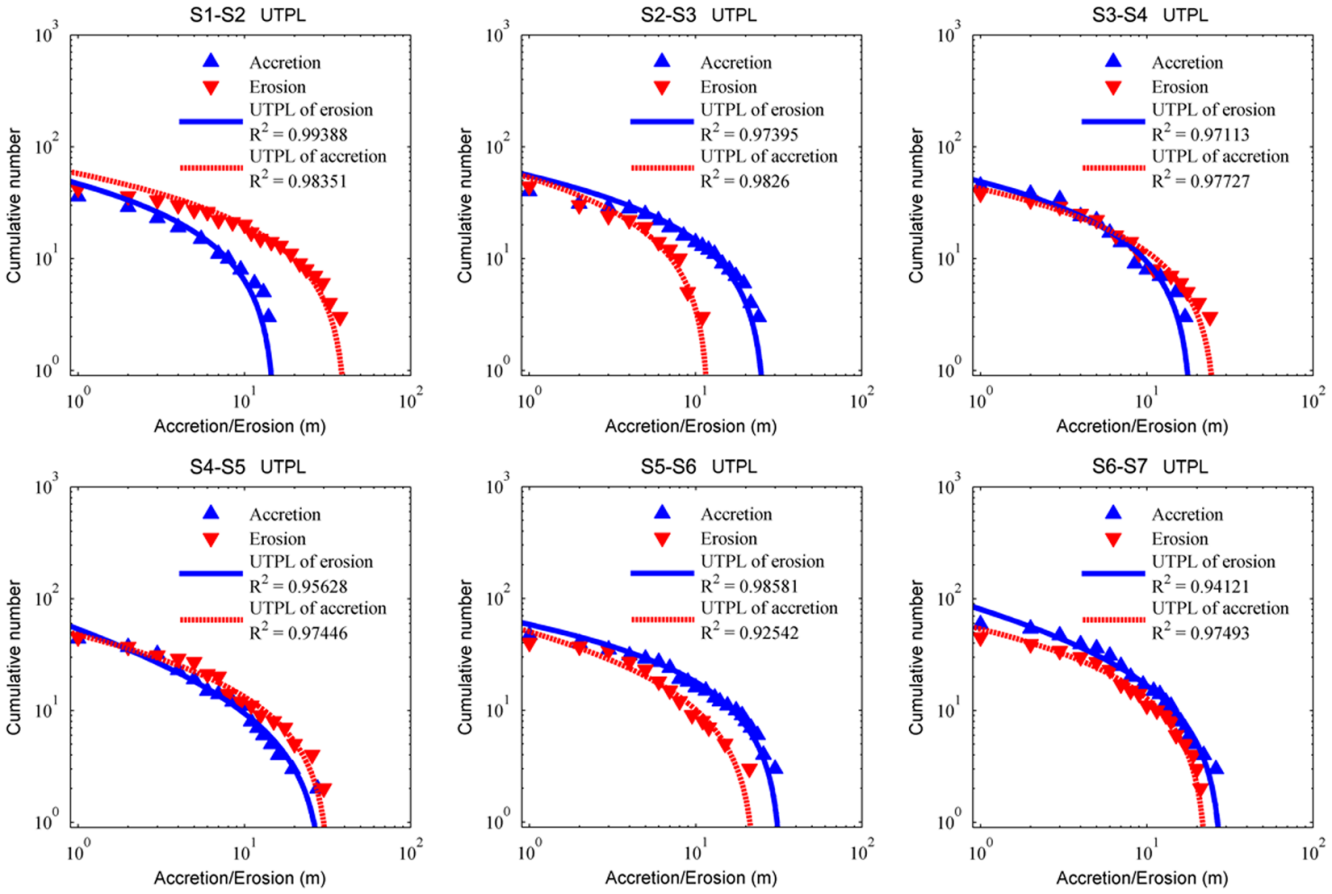
The cumulative distributions of the amounts of shoreline accretion and erosion during the surveys. The plotted points are statistical data based on surveys, which were fit with upper-truncated power law (curved lines); R^2^ is the square of correlation coefficient for the data points and upper-truncated power law.

**Figure 6:**
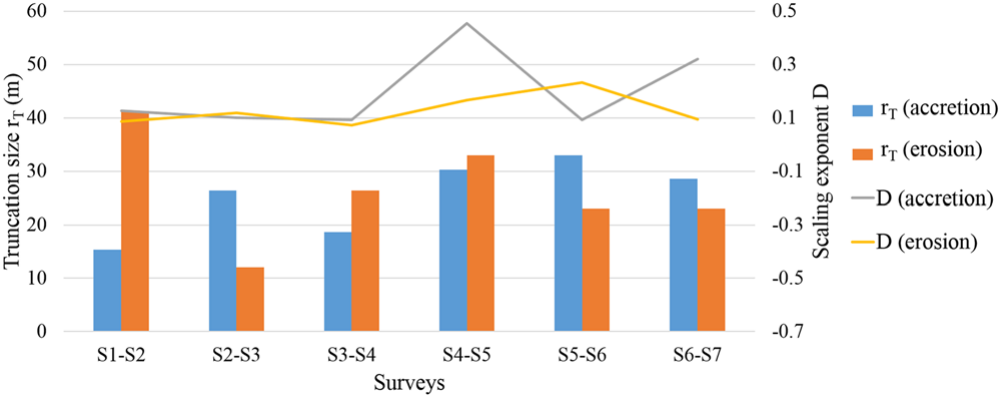
The parameters *r*_*T*_ and *D* of the UTPLs for the cumulative distributions of the amounts﻿ of shoreline accretion and erosion during the surveys.

As shown in Fig. 5, UTPL fits well with the cumulative distribution of shoreline erosion/accretion, demonstrating the existence of an upper limit (*r*_*T*_) of erosion/accretion for a point on the shoreline. The scaling exponent *D* considers the fractal dimension of the shoreline change in the cross-shore direction, as opposed to the power-spectral exponent *β*, which is used to describe the alongshore shoreline change (equation (8)). In UTPL, -*D* is the slope of the log-log plot of the characteristic size and corresponding cumulative number of objects; larger *D* values indicate a higher proportion of smaller objects. The two parameters derived from the UTPL relations in Fig. 5, *r*_*T*_ and *D*, describe the distribution of the horizontal movements of the shoreline during surveys (Fig. 6).

The largest amount of erosion and smallest scale of accretion both occurred in S1-S2 (Fig. 6), which is not surprising because a series of very strong storms affected the study area during this period. The smallest amount of erosion occurred over the time period of S2-S3, making it unlikely that the beach was further eroded in S2-S3 after the severe erosion in S1-S2. For the other period, S6-S7, which is not affected by a storm, the upper limit of erosion is close to the upper limit of accretion, which is twice as much as that of S2-S3, suggesting that the upper limit of erosion had the potential to be much higher than that of S2-S3. Thus, the space for shoreline erosion was reduced to a certain limit during S1-S2. However, the largest scale of accretion did not occur in S2-S3 rather in S5-S6, indicating that the shoreline had suffered from serious erosion due to a series of storms and did not fully recover over the next six months (S2-S3). Generally, the upper limits of shoreline erosion are larger than those of accretion during the periods with storms, but during other times, the opposite is the case; only the *r*_*T*_ of the shoreline erosion/accretion size in S5-S6 does not conform to this general rule. In this case, there are several points to note: 1) during the previous period, S3-S5, typhoons Rammasun and Kalmaegi caused strong shoreline erosion, which is the prerequisite for the large amounts of shoreline recovery observed in the period S5-S6; 2) tropical storm Kujira, which occurred during the period of S5-S6, was relatively weak (Table 1), and this relatively calm weather provided the circumstances for large-scale shoreline recovery and relatively limited erosion; 3) as with shoreline erosion, shoreline accretion is not without a limit. Although there was no tropical cyclone during S6-S7, the upper limit of the amount of accretion in this period did not have to be the highest because some shoreline recovery had occurred before the period of S6-S7, and thus, the space for shoreline accretion was reduced. It is reasonable to expect that after a strong shoreline erosion, the magnitude of the shoreline accretion is also greater, but there is an obvious gap between *r*_*T*_ (erosion) of S1-S2 and *r*_*T*_ (accretion) of S2-S3. However, the difference in the upper limits of the shoreline erosion amounts between the storms series and a single storm is not very clear because the last storm that occurred in S1-S2 was the most powerful one during our surveys (Haiyan, Table 1), which is likely to cause larger erosion than the *r*_*T*_ (erosion) of S4-S5. In summary, the severity of shoreline erosion caused by a series of storms may not be much higher than that caused by a single storm, but the full recovery of the shoreline following serial storms could take a longer time.

Most values of the scaling exponent *D* for the cumulative distribution of the amount of shoreline erosion/accretion during the six survey periods are approximately 0.1, which is quite small compared with the values seen for other natural phenomena^28^. Lower values of *D* mean that there are more locations where the shoreline changes are close to the corresponding upper limit, *r*_*T*_. For periods with small upper-truncated erosion or accretion size, the magnitudes of shoreline changes are relatively small, such that *D* is less influential in determining the size distribution of the shoreline movement. Consequently, a combination of *D* and *r*_*T*_ describes the size and distribution of the shoreline changes in each period. In S1-S2, severe shoreline erosion is widely distributed, with shoreline changes of up to 41.3 m, while shoreline accumulation is limited to less than 15.4 m. In S2-S3, despite the magnitude of shoreline accumulation events being no larger than 26.4 m, a considerable number of the accumulated shoreline events are close to this upper limit. In S3-S4 the erosion magnitudes of many coastal areas are close to 26.4 m; the upper limits of accretion and erosion in S4-S5 are greater than 30 m, but only a few sections of shoreline show accretion close to this level. In S5-S6 the erosion of the shoreline is no more than 23.1 m and the erosion over the majority of the shore is relatively small, but the shoreline accretion during this period is close to 33 m and densely distributed along the coast. In S6-S7, the shoreline also have high magnitude accretion events, but most of the changes in the shoreline are small.

## Discussion

The boundary between the continent and the ocean is constantly changing, and this change can display fractal features, similar to many other natural phenomena. As extreme events, storms have distinct impacts on the fractal properties of shoreline changes. These impacts include but are not limited to the power spectrum of shoreline changes under the influence of storms deviating from the power-law relationship in a specific scale range (from 15 km to 25 km in this case study) via a local reduction in slope and the upper limit of the horizontal shoreline erosion caused by a storm generally being greater than that of accretion. The fractal properties of this change have the potential to identify whether the shoreline change is influenced by the storm.

However, being affected by the sequences and intensities of storms, the storm-induced shoreline changes differ with each storm; consequently, the investigated shoreline changes under the influences of various storms are slightly different from each other in their fractal characteristics (e.g., *r*_*T*_ (erosion) < *r*_*T*_ (accretion) for S5-S6). After the storm, there is usually a recovery of the eroded beach, and the magnitudes and distributions of shoreline recovery tend to balance out the storm-induced shoreline changes^49^. Therefore, shoreline changes under fair weather conditions may also show features similar to those seen after storms (e.g., the power spectrum of the shoreline change in S2-S3), especially after serial storms or a powerful storm, which both cause considerable shoreline changes. As stated above, the wavelet analysis of the alongshore shoreline changes and the UTPL fitting of the cross-shore shoreline changes provide multifaceted information about the overall shoreline movement, which vividly depicts the variations in the shoreline advances and retreats on different spatial scales over different time periods. Therefore, when using the fractal theory on the shoreline changes, a combination of wavelet analysis and number-size distribution studies are required to analyse the different dimensions of shoreline changes (alongshore and cross-shore) and arrive at accurate conclusions.

The intervals between the time of the storms passing Hainan Island and the time of the following surveys were not uniform, which might have differences in the fractal characteristics of the investigated shoreline changes. There are two main reasons for this effect. First, after the storms, especially for the coast sections with severe erosion, the beach may gradually return to its pre-storm state; the degree of recovery is related to the length of time since the event^50, 51^. Second, in addition to the strong influence from storms around the island, waves, tides and other dynamic processes continue to shape the coast, and the intensities and trends of these processes are not considered in this study. In addition, these processes may vary across different time periods. However, the analysis results of the survey data agree well with expectations, that is, the shoreline changes caused by storms share similar fractal properties, which differ from those seen under fair weather conditions. It can also be concluded that the fractal properties of the shoreline changes caused by storms have some temporal continuity.

**Figure 7:**
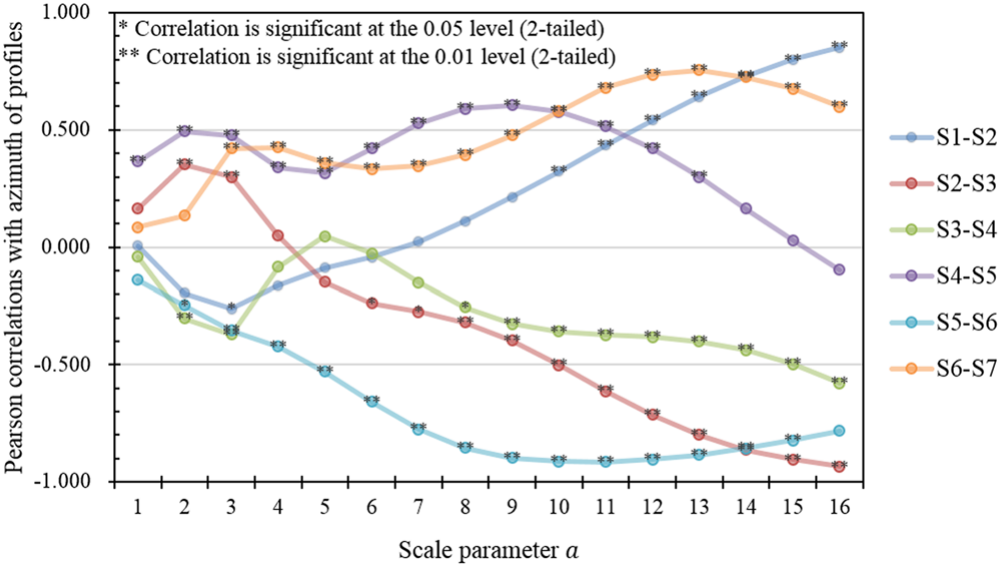
Pearson correlation of the WCMVs between the shoreline changes and azimuth of the profiles from HNJ to YGZ.

As demonstrated by the wavelet analysis, the strongest patterns of the shoreline changes induced by the storm are the fluctuations of the alongshore patterns at a scale of approximately 30 km (fluctuation of the wavelet coefficients in Fig. 4(a) with a scaling parameter a = 3, at which the scale of the WCMV reached a peak). Furthermore, the positions of the alongshore units are relatively fixed, as the wavelet coefficient is always equal to zero at the same or a similar position along the shoreline, especially along the southeastern coast (approximately 140~315 km from HNJ, Fig. 4). Positions where the wavelet coefficients are equal to zero often appear near headlands. This indicates that the open coasts between the two adjacent headlands are affected more by storms than the coasts in the vicinity of the headlands. To assess the correlation between the shoreline changes and the shape of the coastlines, the azimuths of the investigated profiles, which can represent the orientation of the local shorelines, are analysed using a wavelet transform described in equations (4) through (6), and the Pearson correlation coefficient of the WCMV between the azimuth sequence and shoreline changes is calculated at different scales (Fig. 7). For the scales of the alongshore effects of storms (3 ≤ *a* ≤ 5), the correlations between the azimuth sequence and shoreline changes are weak, with absolute values of the correlation coefficients smaller than 0.5. Although the correlations are significantly strong for some larger scales of each shoreline change, no obvious relationships between the variations of the correlations and the scales are observed. This characteristic agrees with the power spectra of shoreline changes on large scales. In addition, many other factors may affect the expressions of the controls of the underlying geological conditions on the shoreline change, including the scales and tracks of storms. The underlying processes of how storms reshape shorelines on particular scales need discussed further with more detailed evidence.

In studying fractals, a power law is often interpreted in terms of self-organization. Self-organization is spontaneous and is not controlled by an external agent, allowing for the formation of scale-free structures (or overall order) through internal interactions. The changes of shorelines are complex nonlinear processes which involves numerous factors with observable variations, and the study of the fractal properties can provide information about the internal characteristics and relationships responsible for shoreline change. According to the power-law behaviour of shoreline variations, the shoreline changes could be self-organized on scales up to 30 km under long-term fair weather conditions (e.g., shoreline change during S6-S7, in this case study), with weak but steady persistence at this scale. Storms play a disrupting role in the gradual self-organization of shorelines for scales from 15 km to 25 km. More specifically, for a time period affected by storm, the alongshore pattern of shoreline changes is robust on a scale 15 km, while patterns with scales of approximately 20 km are more antipersistent. The shoreline changes affected by storms build on the preceding shoreline conditions, including the effects of previous storms and beach recoveries after these storms. During the beach recovery periods, the shoreline changes can exhibit similar fractal properties as those observed following storms, e.g., the power-law behaviour of the shoreline change during S2-S3 is interrupted on scales of 15 km to 25 km. Based on our statistical analysis, the storm-induced shoreline changes are weakly correlated with the configuration of the coastline. However, the temporal scales and the spatial resolutions in this study have some limitations. Further fractal-based studies of datasets with higher spatial and temporal resolutions and longer run times may lead to more concrete conclusions.

## Materials and Methods

### Shoreline change data

In this study, the beach profiles were measured using a RTK-GPS (Trimble Navigation Limited, U.S.A.). The positioning accuracy of the measuring instrument is 2 cm + 2 ppm (× baseline length) horizontally, and 3 cm + 2 ppm (× baseline length) vertically. The distance between the base station and the measuring area is controlled to be within 15 km; therefore, the measuring accuracy is ensured to be less than 6 cm, which is acceptable compared with the magnitudes of the variation of the observed shoreline position. Profiles are measured perpendicular to the local shoreline, from the datum points backshore down to low tide level. The latitudes and longitudes (WGS84 coordinate system) of each profile were recorded in the first survey and then precisely located in the subsequent surveys. To avoid the direct influence of coastal spits, inlets and coastal structures on the higher levels of the beach, the mean sea level (MSL, 0-m contour) is taken as the representative shoreline position^52^. The datum point for each profile is identical between the surveys and has a fixed geographical position. The horizontal distances from the datum points to the measured points on the profiles are calculated by ArcMap [10.1] (http://www.esri.com/) using their latitudes and longitudes. Then, we get a two-dimensional beach profile with an elevation and distance from the datum point at the backshore, hence the distance between the datum point and MSL with an elevation equal to 0 m can be easily and accurately determined. The differences between the horizontal distances from the datum points to MSL of the two surveys are the shoreline changes from the profiles. Therefore, the shoreline changes used for the wavelet analysis are consistent with the shoreline changes $f(x)$ at the sampling profiles *x* in a sequence of 5 km intervals.

### Wavelet analysis

Wavelet transform is effective for all values of fractal dimensions^48^ and provides information on both the spatial and frequency dependences of a data series. Wavelet transforms have distinct advantages over the traditional Fourier transforms when analysing data series that have discontinuities and sharp peaks and for accurately deconstructing and reconstructing finite signals. The wavelet analysis applied to the shoreline changes detailed in the previous section follows the work of Tebbens *et al*.^29^, and the calculations are performed using the Wavelet Toolbox^TM^ in Matlab R2010a. A filter called the Mexican hat wavelet is given in equation (4):

4 $$\psi (x)=(\frac{2}{\sqrt{3}}{\pi }^{-1/4})(1-{x}^{2}){e}^{-{x}^{2}/2}.$$

The above equation is convolved with the shoreline change $f(x)$, as shown in equation (5):

5 $$W(x,a)={\int }_{-\infty }^{\infty }(\frac{2}{\sqrt{3}}{\pi }^{-1/4})(1-{(\frac{x-x^{\prime} }{a})}^{2}){e}^{-{(x-x^{\prime} )}^{2}/2{a}^{2}}f(x^{\prime} )dx^{\prime} .$$

The scaling parameter, *a*, varies from 1 to 16; the wavelet coefficients $W(x,a)$ for the different scales are then returned. The square of the wavelet coefficient measures the variance of the signal; hence, for the average value over the length of the signal *n*,

6 $$WCMV(a)=\frac{{\sum }_{i=1}^{n}W{({x}_{i},a)}^{2}}{n}$$

denotes the mean variance at the corresponding wavelet scale, which is the power spectrum of the shoreline change^34^. Since the profiles are distributed one after another around Hainan Island, there is no true beginning or end of the shoreline change signal. By using the sequence itself to extend the signal on both sides, the edge effect of wavelet transforms is avoided.

If a space-scale (similar to time-frequency) signal is self-affine, the power-spectral density of the signal will have a power-law dependence on its scale (equation (1)), and the power-spectral exponent, i.e., the fractal dimension, can indicate weak or strong persistent signals^48^. To quantify the persistence of shoreline changes, the relationship between the mean variance of the wavelet transform coefficients, *V*, and the wavelet scale parameter, *a*, is examined according to the method mentioned by Malamud and Turcotte^48^ and Lazarus *et al*.^34^:

7 $$\beta =\frac{gradient(\mathrm{log}\,V)}{gradient(\mathrm{log}\,a)}$$

where *β* is the power-spectral exponent. The operation “gradient” calculates the central difference of the interior data points, and the single-sided differences at the ends of the data series. The relationship between the power-spectral exponent *β* and the fractal dimension *D* is given by Voss^53^ as

8 $$\beta =5-2D.$$

However, for a self-affine fractal with dimension $1\le D\le 2$ and the power-spectral exponent values 1 ≤ *β* ≤ 3, *β* is applicable for the self-affine signals for all values, not just 1 ≤ *β* ≤ 3. This is because *β* is a measure of the strength of the persistence: signals with *β* > 1 are non-stationary and have strong persistence; signals with *β* ≤ 1 are stationary and have weak persistence^48^.

### Brown noise

To validate the fractal properties of the shoreline changes, 1,000 sets of BN are generated and analysed. BN is a classic example of a self-affine time series, and can be generated as the cumulative sum of random moves. In this study, 1,000 sets of BN are analysed separately to obtain the WCMVs and *β* series for each signal and are then averaged by scale parameters to form the wavelet analysis results of BN.

### Upper-truncated power law

As mentioned in the *Introduction* and equation (3), the cumulative number-size distributions for the fractals of natural objects can be fitted by UTPL^28^. In this paper, we count the cumulative number N(*r*) and amount of shoreline change *r* of the erosion and accretion profiles for every investigated period and then fit the UTPL to the cumulative distribution of the shoreline erosion/accretion using a genetic algorithm. The term *r*_*T*_ in equation (3) is the object size when *N(r)* equals zero, which may be larger or smaller than the greatest object size in the data set due to the difference between the integrated number of objects and the scatter fitting function^28^.
